# How does the COVID-19 pandemic affect the personal lives and care realities of people with a schizophrenia spectrum disorder? A qualitative interview study

**DOI:** 10.1177/00207640231156833

**Published:** 2023-03-02

**Authors:** Alexander Kaltenboeck, Filipe Portela Millinger, Sarah Stadtmann, Christine Schmid, Michaela Amering, Susanne Vogl, Matthäus Fellinger

**Affiliations:** 1 Clinical Division of Social Psychiatry, Department of Psychiatry and Psychotherapy, Medical University of Vienna, Austria; 2 Sozialpsychiatrisches Zentrum, Caritas der Erzdiözese Wien, Austria; 3 Department of Psychology and Ergonomics, Technical University Berlin, Germany; 4 Department of Social Sciences, University of Stuttgart, Germany

**Keywords:** COVID-19, pandemic, schizophrenia, psychosis, qualitative study, interview study

## Abstract

**Background:**

The COVID-19 pandemic constitutes one of the greatest recent public crises. This study explored its influence on the lives and care realities of people with a schizophrenia spectrum disorder (SSD).

**Methods:**

Between October 2020 and April 2021, semi-structured in-depth interviews were conducted with 30 volunteers with SSDs receiving inpatient or outpatient treatment in Vienna (Austria). Interviews were audio-recorded, transcribed verbatim and analysed thematically.

**Results:**

Three main themes were identified. First, ‘Pandemic life is deprived, lonely and surreal – though certain aspects can be perceived as positive’. Second, ‘Bio-psycho-social support systems were struck at their core by the pandemic and were left severely compromised’. Last, ‘There is a complex interplay between one’s prior experience of psychosis and the experience of the COVID-19 pandemic’. The pandemic situation affected interviewees in various ways. For many, it led to a drastic reduction in day-to-day and social activities and contributed to an atmosphere of strangeness and threat. Bio-psycho-social support providers frequently suspended their services and offered alternatives were not always helpful. Participants indicated that whilst having an SSD might render them vulnerable to the pandemic situation, prior experience with psychotic crises can also provide knowledge, skills and self-confidence which enable better coping. Some interviewees also perceived aspects of the pandemic situation as helpful for recovering from psychosis.

**Conclusion:**

Healthcare providers must acknowledge the perspectives and needs of people with SSDs in present and future public health crises to ensure proper clinical support.

## Introduction

Since its outbreak in early 2020, the COVID-19 pandemic has led to some of the greatest healthcare, social and political challenges in recent decades. Studies on people’s psychological reaction to this public health crisis have revealed a range of quantitative and qualitative insights in the general population (Bonati et al., 2022; Richter et al., 2021; Zrnić Novaković et al., 2022) and various subgroups (McKinlay et al., 2021, 2022; Mizrak Sahin & Kabakci, 2021), including people with different mental disorders (Burton et al., 2021; Gillard et al., 2021; Tandt et al., 2022). The particular experience of the COVID-19 pandemic situation of people with schizophrenia spectrum disorders (SSDs), however, is yet ill-understood.

Several authors using quantitative approaches have reported that people with schizophrenia have been affected comparatively little by the pandemic in terms of their mental health (Barlati et al., 2021; Caponnetto et al., 2021; Fleischmann et al., 2021), and some have attributed this to a certain preoccupation with intrinsic issues in this population (Barlati et al., 2021). Other authors, however, have found an increase in negative symptoms (e.g. anhedonia, avolition and asociality) during the COVID-19 pandemic (Strauss et al., 2022), suggesting that the pandemic’s influence on people with SSDs might be complex and dependent on multiple, potentially interrelating, factors. A qualitative research approach focusing on the first-person perspective of people with SSDs can be helpful in chartering this complexity. To date, however, there is only a small corpus of qualitative literature available on this topic.

For example, Kotlarska et al. (2021) undertook an interpretative phenomenological study in Poland, where they interviewed ten people with chronic schizophrenia without acute psychotic symptoms. The researchers documented a variety of experiences related to the pandemic situation amongst their interviewees, but in general they seemed to cope relatively well and showed psychological reactions similar to other groups. In another study, Karanci et al. (2022) interviewed 18 people with remitted schizophrenia living in Turkey. In their interview data, the authors identified four different groups of factors relevant to life in the COVID-19 pandemic: burdening, positive and neutral impacts of the pandemic situation, as well as facilitators of coping. A complementary approach to these two interview studies was taken by Lyons et al. (2021) who qualitatively analysed Reddit forum posts, by people self-identifying as having psychosis, discussing various aspects of the pandemic situation. The authors identified five recurrent patterns in the forum posts: declining mental health, changed psychosis experience, personal negative and positive coping experiences, social connectedness and disconnectedness and COVID-19 as a metaphor. Similar to Kotlarska et al. (2021), the researchers highlighted the variability of documented experiences and underlined the fact that psychosis experience might not only be perceived as a vulnerability but also as a strength that supports coping in the pandemic situation.

Currently available qualitative studies on how people with SSDs experience the COVID-19 pandemic have important theoretical and practical implications but are limited by their study populations (e.g. predominantly men as interviewees, often living with family/friends/partner and relatively stable in terms of psychotic symptoms), the contexts in which they were conducted (e.g. specific geographical regions or healthcare settings) and the methodologies they used.

The study presented here aims to complement previous qualitative research efforts to further our understanding of how a pandemic situation can influence the life of people with SSDs. We recruited a clinically, demographically and socially diverse group of 30 interviewees and deliberately aimed for generating clinically/practically relevant insights that could be used to better support people with SSDs in public health crises.

## Methods

### Study design

We used an exploratory qualitative research approach to answer the research question ‘How does the COVID-19 pandemic affect the personal lives and care realities of people with a schizophrenia spectrum disorder?’ Data were collected using semi-structured, in-depth, one-on-one interviews conducted by two academic psychiatrists. Interviews were audio-recorded, transcribed verbatim and analysed thematically in order to identify patterns that were recurrent and/or inherently relevant for the research question. The study was approved by the ethics board of the Medical University of Vienna (reference number: 1546/2020) and all people gave written informed consent prior to their research interview.

### Study participants

Thirty volunteers with a diagnosis of an SSD (i.e. any F2 code in ICD-10, except F21 and F23.0) participated in the study. Recruitment was conducted in inpatient and outpatient facilities at the Department of Psychiatry and Psychotherapy, Medical University of Vienna (MUV) and a local collaborating social psychiatric centre, that is Sozialpsychiatrisches Zentrum, Caritas der Erzdiözese Wien (CEW). Recruitment aimed for clinically, demographically and socially diverse study participants, including people with a relatively recent SSD diagnosis as well as experienced ‘SSD veterans’ and people in different treatment contexts (inpatient, day clinic and outpatient). See Table 1 for participant characteristics and Supplemental Material for study exclusion criteria.

**Table 1.** Overview of study participant characteristics (*n* = 30).

Characteristics	Participant data
Gender (self-identified)	Men: 57%
	Women: 43%
Age (years)	Median: 30
	Minimum: 19
	Maximum: 61
Age at onset of disorder (years)	Median: 21
	Minimum: 16
	Maximum: 31
Duration of disorder (years)	Median: 13
	Minimum: 0,5
	Maximum: 40
Lifetime inpatient treatments (estimated count)	Median: 4
	Minimum: 0
	Maximum: 30
Age at first inpatient treatment (years)	Median: 23
	Minimum: 16
	Maximum: 37
Civil status	Unmarried: 83%
	Married or civil partnership: 7%
	Divorced: 7%
	Widowed: 3%
Living arrangement	Single household: 60%
	Shared household/apartment: 16%
	Parent household: 7%
	Assisted living: 17%
Friendships (estimated count)	1 to 3 friends: 47%
	4 or more friends: 53%
Highest education (completed or started)	School leaving examination / Matura: 67%
	Technical/vocational school: 27%
	Mandatory education: 7%
Income	Vocational disability benefit: 30%
	Rehabilitation benefit: 17%
	Basic benefit: 17%
	Unemployment benefit: 3%
	Retirement pension: 3%
	Part-time job: 10%
	Student: 17%
	No answer given: 3%

*Note*. Percentages are rounded to the nearest whole number.

### Data collection and analysis

Data were collected between October 2020 and April 2021 using individual, face-to-face, semi-structured, in-depth interviews based on a topic guide (see Supplemental Material) developed by the research team. Interviews were conducted either at the Department of Psychiatry and Psychotherapy, MUV or at Sozialpsychiatrisches Zentrum, CEW. Interviews were audio-recorded, transcribed verbatim and analysed using a reflexive thematic analysis approach (Braun & Clarke, 2006, 2013; Terry & Hayfield, 2021). After familiarisation with raw data and initial open coding, a tentative coding framework was established within the research team. All transcripts were then coded with this initial coding scheme, adjusting the framework and re-coding transcripts where necessary. Following repeated team discussions and reviewing of codes and transcripts, tentative subthemes and main themes were developed. These again were repeatedly reviewed, discussed, refined and finally defined and named. See Supplemental Material for reflexive statements of all study team members.

## Results

Three main themes were constructed from the interview data. Each main theme comprises several subthemes which provide analytic depth and nuance. Quotes (translated from German to English) illustrating and supporting individual themes can be found in Table 2.

**Table 2.** Overview of representative participant quotes supporting and illustrating themes.

Main theme	Subtheme	Exemplary quote
Main theme 1: pandemic life is deprived, lonely and surreal – though certain aspects can be perceived as positive	Subtheme 1.1: normal day-to-day activities suddenly break away	*Well, it was very bad for my psyche, because I am an arts and culture person and that all pretty much fell away. And so, my resources became less too and all the events, courses fell away, that I did, and that took away very much, a little, my main pillar.* (Participant 04, woman, 25 years)
		*Well, but because I had not been there for a few months, I forgot, I simply forgot to go there again. So, I somehow lost my relation to it [going to bars].* (Participant 01, man, 33 years)
		*[. . .] because that, how shall I say, that ritualised drinking moved into the home, I drink more.* (Participant 25, woman, 61 years)
	Subtheme 1.2: social interactions are reduced and changed	*[. . .] and I was always alone all the time. Cycling alone, reading alone. All alone.* (Participant 05, man, 19 years)
		*[. . .] especially during the lockdown time, where one realised, one must not shake hands, one must not hug, nothing, no? That’s something you notice. (Participant 13, woman, 52 years)*
		*The steps [to deal with psychotic experiences] were mostly that I socialise with people, no? That was somehow number one. And also now that remains number one. What else? To be honest, that is what always helped the most. Now I try to substitute it by, for example, interacting with people over the internet. That works half-decently. Not well. But better than nothing.* (Participant 07, man, 26 years)
		*Because I had no contact with anyone, I had only telephone contacts and being locked in your apartment all alone with psychosis is, I think, really dangerous.* (Participant 04, woman, 25 years)
		*[. . .] so that was isolation, despair, and all that being lonely, well lonesomeness, uh, sadness. Yes, a lot of negative emotions at once. (Participant 04, woman, 25 years)*
	Subtheme 1.3: the pandemic situation is accompanied by a strange and sometimes threatening mood/atmosphere	*Yes, it is truly a strange time. (Participant 02, man, 22 years)*
		*I alwas felt my heart racing when I entered the subway with a mask. I somehow saw humanity lost. No facial expression, no face.* (Participant 05, man, 19 years)
		*Well, I felt a little burnt out. Just all the time coronavirus. Coronavirus this and this and this, and then that no longer. Well, too much bad news is also not good for my psyche, so then I distanced myself [from the news reports].* (Participant 04, woman, 25 years)
		*Suddenly you hear, you must not shake hands, you must not meet people, like you meet normally, so it was totally unreal and it was so unreal that, I did not know, like, I have experienced such unreal things also in psychoses [. . .]. And suddenly something that unreal happens on the outside, yeah*? (Participant 24, woman, age unknown)
	Subtheme 1.4: there are also positive aspects of the pandemic situation	*But on the other hand, it was also quite pleasant. Because personally I am a person who needs much rest anyway and much space and time for myself. And so, I did not have to explain myself. To others.* (Participant 15, woman, 29 years)
		*Uhm, yeah, I think one has more solidarity, because one fights against one threat, everyone against one threat.* (Participant 16, woman, 28 years)
		*[. . .] that is maybe the one positive effect, that I get out of the pandemic with considerably more knowledge.* (Participant 07, man, 26 years)
		*Because one does not have to go there oneself, but it [the prescription] gets sent via fax, it’s somewhat more comfortable.* (Participant 28, man, 25 years)
Main theme 2: bio-psycho-social support systems were struck at their core by the pandemic and were left severely compromised	Subtheme 2.1: professional bio-psycho-social support systems are no longer available in the usual form	*Well, nothing was open. The day clinic was closed, my psychotherapist, I was only allowed to phone her, and the psychiatrist, I think I was also only allowed to phone at times. It was quite difficult.* (Participant 12, man, 22 years)*These are painful conditions and I feel left alone.* (Participant 19, man, 57 years) *[. . .] I was still seeing my psychiatrist on March [redacted], and I also told her I’m hypomanic and I feel bad, and she then said, she told me on the phone, that there are supply shortages for lithium and that totally pulled me down and totally destroyed me, because you can’t say it like that. Then I thought to myself, how am I supposed to get the lithium? Whether I can filter it out from urine again or something. I’ve already had various ideas.* (Participant 16, woman, 28 years)
		*No, I didn’t stop it, it was just that I was overdue with the depot shot. And I should have got it, but the GP didn’t come and give it to me, yeah.* (Participant 11, woman, 24 years)
		*I could only make phone calls [during inpatient treatment], but when you [are] that psychotic or that depressed, then it was very difficult for me too.* (Participant 16, woman, 28 years)
		*Well, with visitor rules I think it’s a shame and I wish it [having visitors] would be possible more often. I don’t know if I want to go so far as to say that I think it’s especially important for psychiatric hospitals, the visiting, I think I’d go that far.* (Participant 28, man, 25 years)
		*We [people receiving psychiatric inpatient treatment] always had to stay in our room and it was indeed very hard, because this was like ultra-lockdown.* (Participant 16, woman, 28 years)
		*Well, previously we [people receiving psychiatric inpatient treatment] used to hug and so on, comfort each other, and now it’s really. . . well, not done. You keep your distance. And yes, that is there, that is that, which has changed.* (Participant 30, woman, 28 years)
	Subtheme 2.2: alternatives to established bio-psycho-social support systems remain compromises	*But I believe, a telephone call or a video conference cannot replace a meeting in person. It can be the solution temporarily, that one says ‘okay for now it [meeting in person] doesn’t work, at least let’s do phone calls’, but simply to replace it, that does not work. (*Participant 24, woman, age unknown)
		*[. . .] the telephone offer is actually not even something I want, because when I come here in person, then I can at least go outside.* (Participant 07, man, 26 years)
	Subtheme 2.3: interaction with the treatment team changed in the pandemic	*[. . .] I get to know a person, yeah, now you for example, with a mask and I simply don’t know at all what you look like as a whole face and when you then take off the mask, then there’s a completely new human in front of me and then sometimes I get a bit scared, because I don’t know how to deal with the situation now.* (Participant 04, woman, 25 years)
		*[. . .] it was all so over the top, all the protective measures, they looked like Martians.* (Participant 16, woman, 28 years)
		*And he [the psychiatrist] was, I think, simply overwhelmed or overburdened. [. . .] I remember a conversation I had with him. There he was, well, during the session someone was constantly calling him, because the hospitals weren’t admitting any patients. And that has changed the quality [of the session] a bit indeed.* (Participant 15, woman, 29 years)
		*Personally, I felt nervous, because I have the feeling that if I spend time here with a physician, then I’m taking up time that could be used for someone who is really sick, right? Although I’m really sick, I know that.* (Participant 07, man, 26 years)
Main theme 3: there is a complex interplay between one’s prior experience of psychosis and the experience of the COVID-19 pandemic	Subtheme 3.1: prior experience with personal psychotic crises is a source of knowledge, skills and self-confidence which can help in dealing with the pandemic situation	*I believe that due to my mental illness, well, mental crisis, which I had, that I have somehow become more steadfast. So, things don’t knock me over that easily anymore.* (Participant 12, man, 22 years)
		*[. . .] it may be that my experience[s] with let’s say isolation have led me to somehow take it easier now.* (Participant 06, man, 51 years)
		*[. . .] go out a bit, that helps, or watch videos, that helps, or the numbers [of COVID-19 cases], only watch the news when things get better. Not when it’s bad. But when things get better.* (Participant 02, man, 22 years)
		*[. . .] and don’t look into conspiracy theories, because otherwise you feel insecure, you panic, you feel watched, you become paranoid. That doesn’t make it any better.* (Participant 02, man, 22 years)
	Subtheme 3.2: a schizophrenia spectrum disorder can entail a certain vulnerability towards aspects of the pandemic	*Well, I think we [people with SSDs] had it harder. So, I don’t think that this [the COVID-19 pandemic] is a situation that you can compare with that [psychosis]. Absolutely, no way. Certainly not, no.* (Participant 09, man, 25 years)
		*Because I generally already have difficulties going outside and with these circumstances it has become even more difficult.* (Participant 27, man, 37 years)
		*[. . .] and of course I totally followed the corona thing, so I read up on virology. The Latin name for corona, coronae in Latin, means ’the listeners’. I interpreted that and somehow thought that this is now like a Ruvuks [sic] Cube, so it’s like a trap or a test of the universe, that you have to solve now and that you can also solve in a spiritual way. And then at some point I imagined I could do magic.* (Participant 16, woman, 28 years)
		*I’m very glad that I don’t have psychosis now, because then I would probably very much, maybe, misunderstand or misinterpret it [the pandemic situation], the whole thing, yes.* (Participant 28, man, 25 years)
	Subtheme 3.3: the pandemic situation – under certain circumstances – can be experienced as helpful and healing	*[. . .] then there was the lockdown, and I just had a quiet, well, dedicated time, where I could isolate, which was very profitable for me. Because I really got permission, now I am allowed to curl up at home, I am allowed to briefly deal with it [psychosis] at home, and I also did not have all that pressure, that I miss out on something outside.* (Participant 04, woman, 25 years)
		*Well, in total, I believe, the [COVID-19] crisis was helpful for me, especially the experience that others too can, how shall I say, become victims of an irritation, and not just I, yeah. (*Participant 25, woman, 61 years)
		*So, there was a little bit of a connection [between people with psychosis and those without], maybe, because of that [the pandemic situation], or an understanding that emerged through it. (Participant 24, woman, age unknown)*
		*Yes, because I [have] the feeling that everyone sticks together and before it just wasn’t like that. (Participant 17, woman, 36 years)*

### Main theme 1: Pandemic life is deprived, lonely and surreal – Though certain aspects can be perceived as positive

Interviewees reported a considerable negative impact of the COVID-19 pandemic situation on their life. The most significant changes were reported with regards to a loss of everyday activities and social interactions. For some, these losses were tied to the sudden non-availability of bio-psycho-social support systems (also see main theme 2), particularly when these had functioned as the sole source of day-to-day structure and social contact prior to the pandemic. Participants also described a negative atmosphere/mood associated with the pandemic situation, characterised from surreal/strange to dangerous/threatening. Nevertheless, there were also certain aspects of the pandemic situation that were perceived as positive, for example reduced day-to-day and social demands, a chance to deepen human connections and acquire new knowledge and improvements in telemedicine.

#### Subtheme 1.1: Normal day-to-day activities suddenly break away

Most study participants reported a loss of usual day-to-day activities, for example visiting restaurants, bars and coffeehouses, physical exercise or engaging with art, culture and religion. This was frequently described as a reduction in the richness and colour of one’s life and as a loss of an important strategy for maintaining mental stability. Notably, some interviewees indicated that everyday activities were lost in the long-term, even if engaging in them was only temporarily hindered by the pandemic situation. In parallel to the reduction of day-to-day activities, increased recreational use of substances (alcohol, nicotine and other drugs) and food intake was observed.

#### Subtheme 1.2: Social interactions are reduced and changed

Participants reported a significant change in social interactions, both quantitatively and qualitatively. Social interaction was reduced (or stopped completely) and shifted to means of communication where physical distance was kept. Newly adopted ways of interacting, for example primarily using video/audio calls or chat services, were often deemed a temporary compromise, not a suitable long-term alternative. Moving-away from known and trusted ways of communicating became challenging for some interviewees, especially when such interactions had previously served as a strategy to manage psychotic experiences (e.g. by using input from friends/family as a trustworthy point of reference against which delusional ideas can be checked). Although, in general, interviewees reported following infection prevention measures, some indicated that, at times, they deliberately ignored physical distancing rules, since they considered face-to-face interaction too important for their mental wellbeing to give it up completely. The changes in social interaction during the pandemic situation gave rise, at times, to strong negative feelings of being isolated and lonely.

#### Subtheme 1.3: The pandemic situation is accompanied by a strange and sometimes threatening mood/atmosphere

Some interviewees reported noticing a peculiar atmosphere/mood, particularly at the beginning of the pandemic, which they described along a continuum ranging from strange and surreal to dangerous and threatening. To them, the world had suddenly changed in a peculiar way and there was a sense of impending doom. Some also worried about the possibility that, without their knowledge, they could be infected with SARS-CoV-2, thereby unintentionally harming others. This atmosphere/mood of strangeness and danger seemed to be fuelled by the adoption of public infection prevention measures, news reports about the local spread of COVID-19 and increasingly heated political controversies. Some also perceived the constant media coverage of the pandemic as exhausting and at times overwhelming, so they deliberately avoided news about COVID-19. Participants also indicated that aspects of their pandemic experience resembled previous experiences of a psychotic break. Interviewees furthermore observed growing tension (sometimes even paranoia) with regards to the pandemic in their social circles. One participant (Participant 27, man, 37 years), showing compulsive scratching, also observed that the pandemic situation caused increased hostility in the public towards ‘odd’ behaviour, feeling that others would interpret such behaviour as a possible sign of COVID-19.

#### Subtheme 1.4: There are also positive aspects of the pandemic situation

Participants also identified positive aspects linked to the pandemic situation. Some described it as a chance to withdraw from tiresome and stressful demands of day-to-day and social life without having to explain themselves or refer to their SSD diagnosis. Especially when psychotic symptoms aggravated and mental stability seemed at risk, the pandemic served as a convenient social excuse to retreat and recover (also see subtheme 3.3). Interviewees also saw a chance for deepening interpersonal relationships, acquiring new skills (e.g. learning a language or taking up a new hobby) and contemplating one’s situation during the pandemic. Some also suggested that they found it easier to leave their home because public spaces were less crowded. Finally, the pandemic led to welcomed changes in medical care (e.g. availability of electronic prescription services).

### Main theme 2: Bio-psycho-social support systems were struck at their core by the pandemic and were left severely compromised

Study participants highlighted that the pandemic situation had a significant effect on the bio-psycho-social support systems on which they normally relied. For many, professional support (including specialist psychiatric outpatient care, psychotherapeutic support, inpatient psychiatric treatment, psychiatric day care, self-help groups and day-structuring facilities) was no longer available in the usual form, resulting in the sudden, unexpected loss of an important safety net. Where alternatives were set up, they were frequently appreciated but also seen as temporary makeshift substitutes rather than proper long-time replacements. Further complicating this situation, some interviewees felt that the way their care team interacted with them had changed in the pandemic situation.

#### Subtheme 2.1: Professional bio-psycho-social support systems are no longer available in the usual form

Interviewees reported that bio-psycho-social support systems, on which they had previously relied, suddenly became unavailable in their usual form. Some support systems (e.g. psychiatric day care centres) suspended their services altogether at the outbreak of the pandemic. Other systems were replaced by makeshift substitutes with reduced face-to-face contact (e.g. psychiatric consultations via telephone calls, psychotherapy via videochat). For some participants, these developments made it harder to get adequate support in dealing with their SSD. Difficulties in accessing bio-psycho-social support systems were especially serious for people who developed an acute psychotic crisis over the course of the pandemic or who did not have any social contacts outside their usual support system.

Some study participants also reported that, due to the pandemic, their established psychopharmacotherapy suddenly became unavailable, leading to worries and anxiety. One study participant (Participant 11, woman, 24 years) reported that, because she had developed a fever and her primary care physician suspected she might have COVID-19, there was a considerable delay in the administration of a long-acting injectable antipsychotic. As a result, she developed severe psychotic symptoms and required acute psychiatric hospitalisation.

Study participants who received inpatient psychiatric treatment or who lived in therapeutic living communities during the pandemic also reported multiple difficulties. Most notably, visits from external people were limited or not allowed at all. This was perceived by many as a significant restriction of personal and social life and deemed disadvantageous for their mental health and recovery from psychosis. Similarly, therapeutic leave during inpatient treatment was also restricted and participants reported that, in some hospitals, they were not even allowed to leave their room due to COVID-19 containment rules. Some interviewees also observed that peer interaction and support amongst inpatients was negatively affected by the pandemic situation.

#### Subtheme 2.2: Alternatives to established bio-psycho-social support systems remain compromises

Interviewees described that many of the bio-psycho-social support services on which they had previously relied, were adapted to suit infection prevention requirements (e.g. psychiatric assessments and psychotherapy being done via telephone rather than in person). In general, participants showed a certain relief and gratitude that, given the situation, these alternative services were upheld. Nevertheless, on a larger scale, these offers were frequently deemed only a temporarily acceptable compromise and not a long-term solution. Participants gave different reasons why alternative services were not (fully) satisfying to them. These included a lack of direct interpersonal contact, discomfort arising from the use of personal protection equipment (PPE), and a lack of motivation to leave one’s house when no face-to-face meeting was scheduled.

#### Subtheme 2.3: Interaction with the treatment team changed in the pandemic

Participants also attributed to the pandemic situation a significant change in the way members of their bio-psycho-social support teams interacted with them. Professional caregivers were suddenly seen using protective measures in face-to-face communication (e.g. face masks, face shields, gowns, communication only behind a glass pane), which, at times, caused considerable problems for interviewees. Some reported, for example, difficulties in interpreting facial expressions and emotions in people wearing face masks, or – especially in the case of acute/emergency psychiatric treatment – in building trust towards people wearing heavy protective gear. Participants also described that their treatment team seemed, at times, distracted or even overwhelmed by the demands of the pandemic situation. Some thought that the support they received was therefore compromised in quality compared to pre-pandemic times. Some participants also expressed worries about their caregivers’ wellbeing in these demanding times, as well as feelings of being ashamed for requesting medical attention in an already difficult situation with stretched resources.

### Main theme 3: There is a complex interplay between one’s prior experience of psychosis and the experience of the COVID-19 pandemic

Study participants suggested that there is a complex relationship between having personal experience with psychosis and living through the COVID-19 pandemic situation. On the one hand, previous psychotic crises were seen by interviewees as a source of transferable knowledge, skills and self-confidence that were useful for navigating the pandemic situation. On the other hand, having an SSD was also perceived as a vulnerability, putting people at increased risk for distress and mental ill-health during the pandemic. Finally, the COVID-19 situation itself was – under the right circumstances – seen as an opportunity for recovery and healing from a psychotic crisis.

#### Subtheme 3.1: Prior experience with personal psychotic crises is a source of knowledge, skills and self-confidence which can help in dealing with the pandemic situation

Several interviewees reported that having personal experience with psychotic crises proved advantageous for them in dealing with the COVID-19 situation. Specific aspects of the pandemic experience – for example social isolation, sudden upheaval of one’s personal and social environment, a sense of impending doom – resembled prior experiences related to psychosis. Participants indicated that by having lived through psychotic crises they had acquired transferable knowledge and skills which allowed them to confront these challenges posed by the pandemic with a certain sense of familiarity and self-confidence. One point where the role of previous experience with psychosis became especially clear was with regards to conspiracy theories. Especially participants who had personal experience with paranoid ideas, identified engagement with such theories in a pandemic situation as particularly dangerous.

#### Subtheme 3.2: A schizophrenia spectrum disorder can entail a certain vulnerability towards aspects of the pandemic

Whilst several participants indicated that their experience with psychosis helped them in dealing with the pandemic situation, others highlighted that having an SSD put them at an increased risk for mental ill-health and made coping more difficult. Even those interviewees who identified strengths derived from past psychotic experiences also noted potential vulnerabilities with respect to the pandemic situation. One risk that was mentioned repeatedly, was that the COVID-19 situation could aggravate symptoms of schizophrenia, especially negative symptoms, such as social withdrawal and self-isolation. Participants also identified the possibility of the pandemic triggering and influencing positive symptoms, for example, delusional ideas or hallucinations. For some, the pandemic situation posed a significant risk of tipping back into full-blown psychosis.

#### Subtheme 3.3. The pandemic situation – under certain circumstances – can be experienced as helpful and healing

Especially people who had experienced a first episode of psychosis, or an aggravation of a pre-existing SSD immediately before or during the first weeks of the pandemic, underlined that they appreciated the temporary slowing-down of everyone’s life. What seemed to be particularly helpful to them was the knowledge that they could withdraw from social interactions safely, without having to worry about damaging relationships or missing out on important developments in their circle of friends. Another aspect of the pandemic situation that was perceived as somewhat healing by interviewees was that now people without any experience of psychosis could relate to certain facets of this condition (e.g. social withdrawal and isolation, a feeling of impending doom or tragedy or the impression of being overwhelmed by the fast-paced development of a situation and a barrage of new and constantly changing information). Interviewees also noted that, especially at the beginning of the pandemic, they observed a new solidarity amongst people, both locally and globally, which some also experienced as healing and wholesome.

## Discussion

We examined the influence of the COVID-19 pandemic situation on the personal lives and care realities of people with an SSD, utilising an exploratory qualitative research design with face-to-face, in-depth interviews and a reflexive thematic analysis approach. Overall, we identified three distinct main themes, each capturing a different aspect of study participants’ experience.

Main Theme 1 describes interviewees’ experience of everyday life during the pandemic. Participants reported a considerable loss of daily routines and social activities due to public infection containment measures. Similar findings have been reported across various populations – including people with psychotic and bipolar disorders (Aminoff et al., 2022; Patel et al., 2022), those with other mental health conditions (Burton et al., 2021; Gillard et al., 2021; Tandt et al., 2022) and different groups without a psychiatric diagnosis (McKinlay et al., 2021, 2022; Mizrak Sahin & Kabakci, 2021). However, loss of day-to-day routines and social activities can be particularly challenging for people with SSDs, where planned daily activities, as well as regular social contact, are often essential pillars to maintain reality testing, mental stability and control negative symptoms. During inpatient psychiatric treatment, the lack of these protective factors might be particularly detrimental (Adorjan et al., 2021; Ma et al., 2020). Thus, it is likely that the risk-benefit-ratio of public infection containment strategies is different for people with SSDs compared to the general population. We believe this aspect should receive considerably more attention in future pandemic management and research.

Study participants also reported sensing a pandemic-related atmosphere/mood, ranging from strange and surrealistic to dangerous and threatening. Qualitatively, this resembles the phenomenon of delusional mood (‘Wahnstimmung’) (Henriksen & Parnas, 2019), which is common in SSDs and sometimes precedes a psychotic crisis. Thus, even when feeling mentally stable, this atmosphere might have led interviewees to worry about the possibility of an incipient relapse into psychosis or might have been an unpleasant reminder of previous (potentially traumatic) psychotic experiences.

Despite the mainly negative effects of the pandemic situation on their life, several participants also identified positive influences (e.g. having spare time to learn something new). Similar observations have been made in various other psychiatric and non-psychiatric study populations (Burton et al., 2021; McCombie et al., 2020; McKinlay et al., 2021; Patel et al., 2022; Tandt et al., 2022).

Main theme 2 highlights participants’ experiences of changes in their help and care systems during the pandemic. As in other studies in people with neuropsychiatric conditions (Aminoff et al., 2022; Giebel et al., 2021), interviewees reported that their support systems suspended services either altogether or limited them to (less helpful) alternatives. For some, regular contact to a help system was crucial for maintaining a structured daily routine and constituted an important – if not the only – source for interpersonal contact and connection. To them, losing these resources caused considerable stress. Loss of support services can also increase the burden on relatives acting as (unpaid) caregivers (Aminoff et al., 2022; Vaitheswaran et al., 2020), however this was not specifically mentioned by our interviewees.

Participants also reported changes in how they experienced their treatment team. Some described, for example, that when clinicians use PPE, it made it harder for them to read facial emotions and build up trust. This parallels findings from recent experimental studies in healthy people, demonstrating that face masks can negatively influence the recognition of facial emotions, as well as other factors important for building a working clinician-patient relationship, for example trust attribution, perceived closeness and re-identification (Grundmann et al., 2021; Kastendieck et al., 2022; Malik et al., 2021; Marini et al., 2021). Whilst it is paramount that we protect people with SSDs against infections with SARS-CoV-2, especially as they might be more vulnerable (Fond et al., 2021; Karaoulanis & Christodoulou, 2021), protective efforts have to be balanced with the potential negative effects of PPE on therapeutic efforts. This applies especially to situations of psychotic crises, where patients already perceive the world as suspicious and odd. Possible solutions might be the use of transparent masks, which seem to better support emotion perception and trust attribution (Marini et al., 2021), or the brief presentation of one’s face from a safe distance. Since in hospital settings the use of PPE will remain an issue in the foreseeable future (as well as in potential future infectious disease outbreaks), the development of strategies to protect oneself and one’s patients against infection without impeding therapeutic relationship building, should be a priority for further research.

Main theme 3 captures a complex interplay of having an SSD and living through the pandemic. A personal history of psychosis was perceived as both, a strength and a burden, in navigating the pandemic situation, paralleling previous qualitative results in people with psychosis (Lyons et al., 2021). On the one hand, participants saw similarities between a personal psychotic crisis and a public health crisis like the COVID-19 pandemic. They indicated that through previous psychotic crises they had gained knowledge, skills and self-confidence which now helped them to better handle the pandemic situation. On the other hand, participants also made clear that having an SSD made them mentally more vulnerable to facets of the pandemic and that they might be at increased risk for aggravation of SSD symptoms or even relapse into full-blown psychosis. Pandemic-associated reductions in day-to-day and social life likely fed into negative symptoms, for example social withdrawal or lack of motivation. This is also in line with findings from a recent quantitative study showing an increase in negative symptoms during the pandemic in people with chronic schizophrenia (Strauss et al., 2022).

The pandemic situation, like other prominent public topics, can also shape the content of positive symptoms (Cegla-Schvartzman et al., 2022; Ovejero et al., 2020). Our study participants described such shaping in various manifestations: as an aggravated feeling of impending doom, as paranoid interpretations of public infection prevention measures, up to a full-blown psychotic break with SARS-CoV-2 and the COVID-19 pandemic being the content of vivid hallucinations and bizarre delusional beliefs.

Finally, interviewees also reported that the pandemic situation provided a chance for resting, recovering and healing in the context of an SSD. Similar reports of ‘lockdown relief’ have also been documented by other authors in different study populations with mental health problems (Gillard et al., 2021; Tandt et al., 2022). Participants furthermore suggested that the pandemic situation allowed people without a diagnosis of an SSD to experience certain aspects of this condition first-hand, thereby potentially increasing future understanding and empathy for those with SSDs. This parallels findings from a study by Lyons et al. (2021), who analysed Reddit discussion forum posts by people self-identifying as having psychosis. These authors also reported a pattern amongst Reddit posts, whereby the COVID-19 pandemic was perceived as a useful metaphor for explaining psychotic experiences.

The results of this study should be viewed in the light of several possible limitations. Interviews were conducted by physicians, potentially influencing what participants chose to disclose and withhold. This might have been especially pronounced when there was a pre-existing doctor-patient relationship between interviewer and interviewee. However, it also could have allowed for a privileged access to information and better insight that would otherwise not have become available. Furthermore, data collection was conducted only until April 2021, thus later phases of pandemic experience were not covered in the interviews.

To conclude, the COVID-19 pandemic had, and likely will continue to have, a significant influence on the personal lives and care realities of people with SSDs. The experience of the pandemic situation in our sample showed similarities to the experience of people with other mental health difficulties as well as those without a specific psychiatric diagnosis. Several aspects were specific for people with SSDs and deserve further consideration in future research. The pandemic situation undermined individual coping strategies and professional support systems. Yet, having a history of a personal psychotic crisis can also help to better deal with a public health crisis like the COVID-19 pandemic. Healthcare providers must acknowledge the fist-person perspective and needs of people with SSDs in present and future public crises in order to ensure proper bio-psycho-social support.

## Supplemental Material

sj-docx-1-isp-10.1177_00207640231156833 – Supplemental material for How does the COVID-19 pandemic affect the personal lives and care realities of people with a schizophrenia spectrum disorder? A qualitative interview study Click here for additional data file. Supplemental material, sj-docx-1-isp-10.1177_00207640231156833 for How does the COVID-19 pandemic affect the personal lives and care realities of people with a schizophrenia spectrum disorder? A qualitative interview study by Alexander Kaltenboeck, Filipe Portela Millinger, Sarah Stadtmann, Christine Schmid, Michaela Amering, Susanne Vogl and Matthäus Fellinger in International Journal of Social Psychiatry
