# Supplementary material for: How does the COVID-19 pandemic affect the personal lives and care realities of people with a schizophrenia spectrum disorder? A qualitative interview study
Source: Int J Soc Psychiatry. 2023 Mar 2;69(5):1239–49. doi: 10.1177/00207640231156833 (PMC9988625; doi:10.1177/00207640231156833)
Supplement: sj-docx-1-isp-10.1177_00207640231156833 – Supplemental material for How does the COVID-19 pandemic affect the personal lives and care realities of people with a schizophrenia spectrum disorder? A qualitative interview study [file sj-docx-1-isp-10.1177_00207640231156833.docx]

# Supplements

## Exclusion criteria for the study

Exclusion criteria for participation in the study were as follows:

(a) impaired ability to understand the interviewers and to interact with them during the interview,

(b) significant language barrier,

(c) known intellectual disability,

(d) known neurodevelopmental condition (e.g., autism spectrum disorder), or

(e) participation deemed potentially harmful to the participant.

## Interview topic guide (as used in German language)

Sentences highlighted in yellow are main questions

*Sentences in italics represent further possible prompts and probes*

1. Einleitung (Aufklärung über die Studie, Einverständniserklärung etc.)
2. **Wie ist es Ihnen in der letzten Woche ganz allgemein gegangen?** [Konversationsstarter]
   - Wie ist Ihre derzeitige psychische Verfassung?
   - In welchem Betreuungssetting stehen Sie zurzeit?
   - Hat sich Ihre Betreuung in den letzten Monaten verändert?
3. **Was denken Sie über das Coronavirus und die Maßnahmen, die eingeführt wurden, um die Ausbreitung des Virus zu verhindern?**

- Wie bedrohlich schätzen Sie das Coronavirus für sich selbst und andere derzeit ein? Hat sich Ihre Einschätzung im Lauf der Zeit verändert?
- Welche Vorsichtsmaßnahmen haben Sie persönlich getroffen?
- Für wie sinnvoll halten Sie die empfohlenen Vorsichtsmaßnahmen (z.B. kein Händeschütteln, Tragen von Masken in Supermärkten, keine Menschengruppen, keine Ausgänge im Krankenhaus etc.)? Hat sich Ihre Einschätzung im Lauf der Zeit verändert?
- Inwiefern empfinden/empfanden Sie bestimmte Maßnahmen (z.B. striktes Zuhausebleiben, kein Ausgang im Krankenhaus) als beschränkend? Hat sich Ihre Einschätzung im Lauf der Zeit verändert?

1. **Wie hat sich Ihr Alltag [im Krankenhaus bzw. zuhause] durch die Coronavirus-Situation verändert?**
   - Gibt/Gab es wichtige Aktivitäten, die Sie aufgrund der Vorsichtsmaßnahmen nicht machen können/konnten?
   - Gibt/Gab es für Sie stabilisierende Aktivitäten, die Sie aufgrund der Vorsichtsmaßnahmen nicht machen können/konnten?
   - In welcher Weise hat sich Ihr Sozialleben durch das Coronavirus verändert?
   - Welche Formen der Kommunikation haben Sie im Rahmen der Coronavirus-Krise genutzt, um mit anderen Menschen in Kontakt zu bleiben? Waren diese Kommunikationsmittel neu für Sie oder haben Sie sie schon zuvor genutzt?
   - Intramural: Wie erleben/erlebten Sie den Kontakt zu Mitpatient:innen während der Coronavirus-Situation?
   - Intramural: Wie empfinden/empfanden Sie die Besuchs- und Ausgangsbeschränkung?
   - Extramural: Wie empfinden/empfanden Sie die die strikten Ausgangsbeschränkungen?
2. **Wie wirkt sich die Coronavirus-Situation auf Ihr psychisches Wohlbefinden aus?**
   - Welche negativen Auswirkungen der Coronavirus-Situation haben Sie an sich bemerkt?
   - Welche positiven Auswirkungen der Coronavirus-Situation haben Sie an sich bemerkt?
   - Haben sich Ihre Symptome durch die Coronavirus-Situation in irgendeiner Weise verändert?
   - Gab es unterschiedliche Phasen bzw. wie waren diese voneinander abgrenzbar?
3. **Wie hat sich Ihre psychiatrische Betreuung durch die Coronavirus-Situation verändert?**
   - Auf welche Weise werden/wurden Sie betreut (z.B. speziell gestaltete Visiten, Telemedizin)? Welche Unterschiede im Vergleich zu vor der Coronavirus-Krise haben Sie bemerkt und wie haben Sie diese erlebt?
   - Wie ist/war Ihre Wahrnehmung des psychiatrischen Personals während der Corona-Krise? Welche Unterschiede haben Sie bemerkt?
4. **Welche Rolle denken Sie spielt Ihre Vorerfahrung mit psychischen Krisen im Umgang mit der derzeitigen Situation?**
   - Inwiefern könnte Ihre Erfahrung im Umgang hilfreich (gewesen) sein?
   - Inwiefern könnte Ihre Erfahrung im Umgang problematisch (gewesen) sein?
5. **Wenn Sie freie Wahl hätten, was würden Sie an Ihrer psychiatrischen Betreuung während der Coronavirus-Situation verändern?**
   - Was könnte/hätte Ihr Behandlungsteam (Psychiater:innen, Psychotherapeut:innen, Sozialarbeiter:innen etc.) tun/können, damit Sie mit der Situation besser zurechtkommen/gekommen wären?
   - Was würde/hätte Ihnen helfen/geholfen die Coronavirus-Situation (noch) besser zu überstehen?
6. **Haben Sie noch weitere Gedanken oder Kommentare, die für uns relevant sein könnten?** [Abschluss]
   - Gab es etwas das Sie bisher noch nicht sagen konnten?
   - Haben Sie noch Fragen?

## Reflexive statements of each study team member

### A.K.

I am a medical doctor specialising in psychiatry and psychotherapeutic medicine. I have previously also completed an interdisciplinary master’s degree (philosophy, psychology, neuroscience, informatics) and a doctoral degree working in the area of cognitive neuroscience. Being a clinical psychiatrist during the COVID-19 pandemic, it became quickly clear to me that—apart from some informal grasp and some vague intuitions—I had no adequate knowledge of how our patients, especially the most vulnerable, were affected by the COVID-19 pandemic. This motivated me to, together with M.F., draft a research project that aimed to fill this knowledge gap. I subscribe to a multi-perspective approach to understanding mental disorder that includes nomothetic and idiographic strategies and is ultimately motivated by the pragmatic wish to alleviate the patient’s suffering and restore mental health and wellbeing. Some of the study participants I knew either from previous clinical encounters or because I served as their doctor at the time when I interviewed them. Whilst I am aware that this can complicate matters in many regards, I believe it also allowed for a privileged access to information that would otherwise not have become available.

### F.P.M.

I’m a psychiatric trainee at the Division of Social Psychiatry of the Medical University of Vienna, currently working at the University Hospital Vienna and the Prison Göllersdorf for mentally ill offenders. While I’m not specialized in psychosis, as I’m at an early stage of my training, I’m particularly interested in the experience of people with psychotic episodes. Within the scope of my work in a forensic institution, I’m constantly confronted with the burden and impact on quality of life of psychosis. A better understanding of psychotic experiences is essential for the improved care or support of affected people and therefore shapes my research interest.

### S.S.

I am a psychologist currently working at an outpatient treatment center of Caritas Vienna for people affected by psychosis. I also worked as a research Assistant at the Clinical Division of Social Psychiatry of the Medical University of Vienna. The focus of my clinical work is to support people with psychotic experiences to improve their quality of life. Working on this study was an opportunity for me to gain a better understanding of the impact that the COVID-19 pandemic had on our patients. My research interest is to gather a better understanding how people with psychosis experience their day-to-day life (including the challenges they face), in order to improve current treatment options.

### C.S.

I am involved in the research presented as a medical anthropologist to assist with methodological issues and questions of qualitative data collection and analysis. I work as a research assistant at the Department of Psychology and Ergonomics at Technische Universität Berlin and the Brandenburg Medical School Theodor Fontane and teach at Humboldt-University Berlin. Having conducted qualitative research for eight years, ranging from fieldwork to interviews in sensitive medical settings such as psychiatric wards, home treatment, psychiatric day centers, but also with breast cancer patients, I contributed drawing from my experience and expertise with qualitative research processes in this field. My dissertation, for instance, focused on the co-production of experiential expertise within the field of professional peer support work in psychiatric care. Thus, I can relate to the setting of this study and its specific challenges. My understanding of qualitative research in healthcare is deeply tethered to reflexive work that requires continuous negotiation of the objects of research and the researcher's position in relation to those objects. It is this perspective, that I have tried to pass on and therefore help the group to oscillate between empirical research and analysis and between position as researcher and as practitioner.

### S.V.

I am a sociologist and methodologist with extensive experience with a wide range of methods of data collection and analysis. My aim is to improve existing methods in social science and developing methods further. Hereby, I have expertise in qualitative and quantitative interview methodologies as well as qualitative and quantitative analytic strategies. The goal of my research is to generally advance the field of research methodology and more specifically create awareness for peculiarities of target groups and methods. As a consequence, tailored data collection methods should be more feasible. My research has given me insights into how reflexivity is crucial for the quality of our research output. I work with different methodologies and techniques and I am aware that I favor constructivist approaches to research methods, considering interviews as social interactions that are highly context dependent. However, I know that not every research question and design aims for an in-depth reconstruction but remains on more manifest level of content. I value discursive exchange in analytic sessions to reflect on different readings of the interview materials to ensure intersubjectivity and communicative validity. It is this attitude that influenced the collaboration when analyzing the data for this study.

### M.A.

I am a psychiatrist and Professor at the Division of Social Psychiatry of the Medical University of Vienna/Austria. The main focus of my clinical, teaching and research activities is on the situation of persons with a diagnosis of schizophrenia and their families and friends. I am a proponent of the concept and practice of ‘Trialogue’, a setting which allows communication and collaboration on equal footing between people with a lived experience of mental health problems, their families and friends, and mental health professionals, generating a specific and independent form of acquisition and production of knowledge. My national and international co-operations with advocacy movements of people with lived experience are informed by the historic convergence of the implications of the recovery concept with the expanding volume of scientific evidence as well as the actual changes of the human rights situation of persons with disabilities regarding support for a self-determined life in the community. My research interests are shaped by the significant value I assign to gaining insight into people’s lived experience. I experienced the COVID-19 pandemic as a very serious impediment on my personal and professional life with at times significant impact on my mental health.

### M.F.

I am a psychiatrist at the Department of Social Psychiatry at the Medical University of Vienna/Austria, with a clinical and scientific focus on improving the care of people with severe mental disorders, most recently those affected by psychosis. In addition to establishing the best available and validated treatment, I see the involvement and understanding of people with lived experience and their friends and families as a key factor for joined success in the development of helpful care/offers. Since my research expertise was mainly in quantitative methods, I on the one hand tried to deal intensively with qualitative research methods before and during the study and reflected my perspectives and my understanding accordingly, and on the other hand strategically/on purpose involved three highly experienced qualitative researchers with different backgrounds from the start of our study (C.S., S.V., M.A.) to achieve the highest possible quality. In my reflexivity as a crucial element of quality assurance I am convinced that my experience in clinical and therapeutic work was and is of great help. In my perception and interpretation, I am influenced by my therapeutic-systemic approach, which recognizes and values the environment, particularly its social interactions and relationships, as decisive factors for the current state. Thus, as I had also been the treating psychiatrist for many of the participants I interviewed, special considerations and reflexions on the influence of this role accompanied the interviews and the analysis of the data. I am aware of several possible problematic aspects, especially the power imbalance, but I have experienced it, also in reflection with the study team, as a great strength, above all, to be able to connect even more directly to the reality of participants through the existing trust and prior knowledge.
